# Postnatal Steroids in Preterm Infants: A Narrative Review Series—Part 3: Impacts on Growth, Neurodevelopment & Nutrition

**DOI:** 10.3390/children13040475

**Published:** 2026-03-29

**Authors:** Phoenix Plessas-Azurduy, Jarred Garfinkle, Marc Beltempo, Caroline Porraccio, Anie Lapointe, Laila Wazneh, Punnanee Wutthigate, Sarah Spénard, Anne Marie Sbrocchi, Marie-Brossard Racine, Wadi Mawad, Tiscar Cavallé-Garrido, Wissam Shalish, Guilherme Sant’Anna, Gabriel Altit

**Affiliations:** 1 Division of Clinical & Translational Research, Faculty of Medicine and Health Sciences, Montreal Children’s Hospital, McGill University, Montreal, QC H3A 0G4, Canada; phoenix.plessas-azurduy@mail.mcgill.ca; 2 Division of Neonatology, Department of Pediatrics, Montreal Children’s Hospital, Montreal, QC H3A 0G4, Canadamarc.beltempo@mcgill.ca (M.B.); laila.wazneh@muhc.mcgill.ca (L.W.); sarah.spenard.med@ssss.gouv.qc.ca (S.S.); wissam.shalish@mcgill.ca (W.S.); guilherme.santanna@mcgill.ca (G.S.); 3 Division of Nutrition, Department of Pediatrics, Montreal Children’s Hospital, Montreal, QC H3A 0G4, Canada; caroline.porraccio@muhc.mcgill.ca; 4 Division of Neonatology, Department of Pediatrics, CHU Sainte-Justine, Université de Montréal, Montréal, QC H3T 1C5, Canada; anie.lapointe@umontreal.ca; 5 Department of Pediatrics, Faculty of Medicine Siriraj Hospital, Mahidol University, Bangkok 10700, Thailand; punnanee.wut@mahidol.edu; 6 Division of Pediatric Endocrinology and Metabolism, Montreal Children’s Hospital, McGill University, Montreal, QC H3A 0G4, Canada; annie.sbrocchi.med@ssss.gouv.qc.ca; 7 Division of Pediatric Endocrinology and Metabolism, Department of Pediatrics, McGill University Health Centre, Montreal, QC H4A 3J1, Canada; marie.brossardracine@mcgill.ca; 8 Department of Pediatrics, Montreal Children’s Hospital, Montreal, QC H3A 0G4, Canada; wadi.mawad@mcgill.ca; 9 Division of Cardiology, Department of Pediatrics, Montreal Children’s Hospital, Montreal, QC H3A 0G4, Canada; tiscar.cavalle2@muhc.mcgill.ca

**Keywords:** postnatal corticosteroids, bronchopulmonary dysplasia, somatic growth, body composition, neurodevelopmental outcomes, skeletal health, precision surveillance, hpa axis suppression, igf-axis regulation, physiology-informed care

## Abstract

**Highlights:**

**What are the main findings?**

- Contemporary low-dose, late-onset corticosteroid (CS) protocols exhibit a risk-dependent relationship with neurodevelopment; while historical high-dose “blast” regimens caused harm, modern targeted therapy may offer neuroprotective benefits for infants at the highest risk of bronchopulmonary dysplasia (BPD) while still posing risks for skeletal demineralization and HPA-axis suppression.
- Systemic CS can trigger a profound catabolic state by suppressing the IGF-axis and inducing intense protein wasting, which shifts growth trajectories toward excessive fat accretion over lean mass and creates a clinical picture that traditional metrics like weight and head ultrasound fail to accurately capture.

**What is the implication of the main findings?**

- Clinicians must move beyond crude measurements toward a multi-modal precision surveillance framework, integrating advanced tools like MRI, General Movements Assessment (GMA), body composition analysis, and Near-Infrared Spectroscopy (NIRS), to detect subtle alterations in tissue quality, brain function, and regional oxygenation while being exposed to CS.
- Management should adopt a physiology-informed, individualized approach that balances pulmonary gains against systemic trade-offs by leveraging multi-modal monitoring tools allowing for risk-stratified decision-making and implementation of specialized strategies to support lean mass recovery.

**Abstract:**

Background: Postnatal corticosteroids (CS) improve respiratory outcomes in preterm infants, but effects on growth and neurodevelopment remain incompletely understood. Methods: This third instalment of a narrative review series builds on physiologic principles to examine systemic CS consequences. Main Findings: We explore the interplay between growth restriction, hypoxia, and neurodevelopmental vulnerability, discussing brain imaging, metabolic disruptions, and HPA axis suppression. Conclusion: This review advocates for a holistic, physiology-informed approach to optimize outcomes by integrating nutritional vulnerability with cardiorespiratory status.

## 1. Introduction

**Figure 1:**
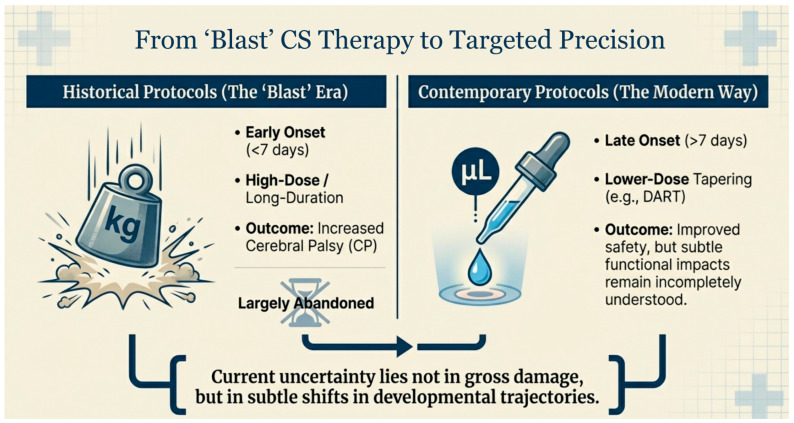
From Historical High-Dose ‘Blast’ CS Therapy to Targeted Precision: The transition from early, high-dose historical protocols to contemporary, lower-dose tapering regimens. Abbreviations: Corticosteroids (CS).

Postnatal systemic corticosteroids (CS) have long been used in extremely preterm infants to treat evolving lung disease and facilitate weaning from invasive respiratory support [1,2,3]. While early high-dose regimens were associated with clear adverse neurodevelopmental and growth outcomes, contemporary practice has shifted toward lower-dose, later-onset protocols aimed at preserving pulmonary benefit while minimizing harm. This evolution from historical high dose “blast” era protocols to modern, targeted precision regimens, characterized by lower doses and later onset, is depicted in Figure 1. Despite these changes, uncertainty persists regarding the true impact of modern CS exposure on somatic growth, skeletal health, gastrointestinal/HPA axis health and neurodevelopment, particularly in the most vulnerable infants born at the lowest gestational ages [4,5,6].

Disruptions to postnatal growth trajectories, especially during critical windows of brain development, have been consistently linked to adverse long-term cognitive, motor, and cardiometabolic outcomes [7]. CS, through their systemic metabolic, inflammatory, and neurobiological effects, may influence these trajectories both directly and indirectly by acting on the other previously mentioned domains (e.g., skeletal health or the HPA axis) [8,9]. However, existing evidence is heterogeneous, often confounded by illness severity, and frequently relying on crude outcome measures (such as weight, length, head circumference, head ultrasound, and later childhood neurodevelopmental endpoints) that may fail to detect subtle but clinically meaningful effects.

Advances in physiologic and neurodevelopmental assessment tools offer an opportunity to refine how CS-related outcomes are evaluated. Techniques such as detailed growth phenotyping (ex. by respiratory disease trajectory/BPD risk and comorbidity profile), bone health monitoring, early functional motor assessments, and advanced neuroimaging may provide greater mechanistic insight than traditional endpoints alone [10]. In this narrative review, we synthesize current evidence on the effects of postnatal systemic CS on growth, skeletal health, gastrointestinal/HPA axis integrity and neurodevelopment in preterm infants, critically examine limitations of existing outcome measures, and highlight emerging tools that may improve future research and clinical decision-making.

## 2. Systemic Impacts: How Postnatal Steroids Affect Growth and Development

**Figure 2:**
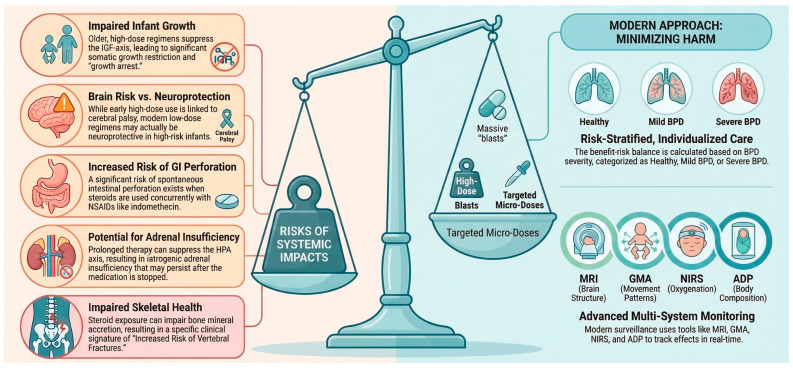
Conceptual Overview of Postnatal CS—Integrating systemic risks with a risk-stratified, individualized framework for minimizing harm. Abbreviations: CS (corticosteroids); IGF (insulin-like growth factor); CP (cerebral palsy); GI (gastrointestinal); HPA (hypothalamic-pituitary-adrenal); MBDP (metabolic bone disease of prematurity); BPD (bronchopulmonary dysplasia); MRI (magnetic resonance imaging); GMA (General Movements Assessment); NIRS (near-infrared spectroscopy); ADP (air displacement plethysmography).

A conceptual overview of the systemic risks associated with postnatal CS and the modern, individualized framework for risk-stratified care and multi-system monitoring is illustrated in Figure 2 providing a visual summary of this delicate balancing act.

### 2.1. Endocrine and Metabolic Mechanisms

Neonatal growth is regulated by a coordinated network of endocrine and metabolic pathways, primarily involving the growth hormone (GH), insulin-like growth factor (IGF) axis, insulin signaling, and thyroid hormone activity [11]. During the fetal period, the maternal–placental–fetal unit provides a tightly regulated supply of hormones such as placental growth hormone (PGH), human placental lactogen (hPL), and IGF-2 [12]. At birth, the sudden dissociation of this unit causes an immediate withdrawal of these placental hormones, forcing the neonate to adapt to extrauterine life [11]. Postnatally, growth is initially driven by a nutrition-dependent pathway where nutrient intake stimulates insulin release, which in turn triggers IGF-1 production. This nutritional control remains the primary driver of mass accretion until the GH-axis matures (typically between 6–9 months of age), at which point GH becomes the main regulator of IGF synthesis.

**Figure 3:**
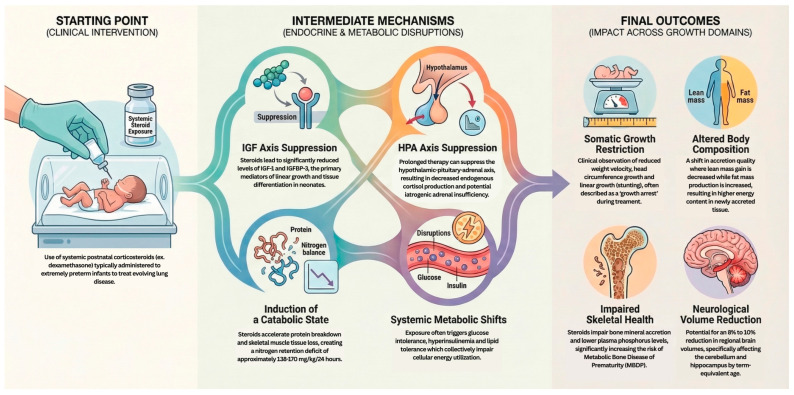
Schematic of Systemic CS Impacts on Growth and Metabolism. Abbreviations: CS (postnatal corticosteroids); IGF (insulin-like growth factor); CP (cerebral palsy); GI (gastrointestinal); HPA (hypothalamic-pituitary-adrenal); MBDP (metabolic bone disease of prematurity); BPD (bronchopulmonary dysplasia); MRI (magnetic resonance imaging); GMA (General Movements Assessment); NIRS (near-infrared spectroscopy).

Exogenous systemic CS significantly disrupt these delicate hormonal trajectories. CS promote a profound catabolic state, characterized by intense protein breakdown and nitrogen retention deficits. High-dose dexamethasone has been shown to cause complete growth arrest by suppressing the IGF axis, specifically reducing levels of IGF-1 and its binding protein IGFBP-3, which are directly correlated with linear growth restriction. Furthermore, prolonged CS exposure can lead to iatrogenic adrenal insufficiency by suppressing the hypothalamic-pituitary-adrenal (HPA) axis, as well as inducing glucose and lipid intolerance. In preterm infants, this hormonal disruption often creates a clinical picture resembling panhypopituitarism, where the lack of growth-promoting hormones combined with high metabolic stress leads to poor longitudinal growth and excessive fat mass accretion relative to lean mass. The mechanistic pathways through which systemic CS exposure triggers a cascade of endocrine and metabolic disruptions, resulting in adverse outcomes across somatic, skeletal, and neurological growth domains, are summarized in Figure 3.

### 2.2. Defining Growth Outcomes Across Domains

To provide a comprehensive overview of these systemic consequences, the following sections examine the specific impacts of postnatal corticosteroid exposure across somatic, neurological, cardiopulmonary, skeletal, and gastrointestinal/HPA axis domains. In this review, ‘growth impairment’ refers to one or more of the following domains: (i) weight gain/weight velocity, (ii) linear growth (length and length velocity), (iii) head circumference growth and (iv) growth quality/body composition (lean vs. fat accretion). Throughout this review, we use the term ‘evolving BPD’ to describe extremely preterm infants with persistent respiratory support needs beyond the first postnatal week who remain at high risk for meeting BPD criteria later in hospitalization. Since BPD definitions and severity classifications have evolved across study eras, we contextualize key findings using study-specific definitions where possible. Lastly, we use ‘early’ to refer to exposure within ≤7 postnatal days and ‘late’ to refer to exposure after >7 postnatal days unless otherwise specified.

#### 2.2.1. Somatic Growth

Studies have associated CS with growth suppression [13]. In 2001, a clinical trial examined the impact of a 42-day tapering dexamethasone regimen versus a repeatable 3-day pulse course on growth and insulin-growth factor (IGF)-axis regulation in 40 preterm infants with chronic lung disease [14]. The findings indicated that higher dexamethasone doses and treatment duration negatively affected IGF-I and IGFBP-3 levels, suggesting that dexamethasone-related growth impairment may occur through IGF axis suppression. This study reported a median total cumulative dose of dexamethasone of 5.3 mg/kg in the pulse group and 7.1 mg/kg in the long duration group, starting at 7 days of life.

Although it remains unclear whether this effect is temporary, cumulative dose-dependent effect on growth impairment has been documented in other studies. In a trial assessing the effects of concomitant growth hormone (GH) therapy in ventilated very low birth weight infants receiving dexamethasone, GH administration did not significantly improve growth outcomes [15]. This study found that complete growth arrest occurred during high-dose dexamethasone exposure, whereas growth in head circumference and weight during lower-dose dexamethasone was comparable to growth after dexamethasone cessation.

In an observational study of patients exposed to different dosages and durations of dexamethasone or hydrocortisone therapy for BPD, decreased growth velocities were observed in the CS treated groups during and shortly after treatment [16]. However, this population was compared to a reference group who had not received CS, which had higher median gestational age at birth, higher birth weight and overall have less serious pulmonary morbidity than the population treated with CS.

Studies have also shown associations between postnatal CS use and decreased weight gain, linear growth, and head circumference development [15,17,18,19]. However, many of these studies were conducted decades ago, utilizing higher cumulative doses, greater daily doses, and early exposure before day 7 of life, often in infants who did not necessarily have significant inflammatory lung disease progressing toward BPD.

A recent multicenter cohort study reported the impact of postnatal systemic CS [20] on overall growth and described no significant effect. However, preterm infants who had received postnatal CS but were not invasively ventilated at 28 days had decreased head growth at hospital discharge. Furthermore, information regarding total cumulative dose of postnatal CS was not provided.

The clinical management of infants with BPD is frequently complicated by an intrinsic hypermetabolic state and subsequent growth failure, which are often firmly established within the first month of life [21,22]. Research indicates that these patients experience demonstrable reductions in both muscle and fat accretion, accompanied by a profound decrease in overall growth velocity [18]. A multicenter retrospective cohort study of infants born <30 weeks’ gestation between 2015–2018 found that increases in body mass index (BMI) z score from birth to 36 weeks CGA were associated with higher odds of BPD [23]. Additionally, although infants had similar caloric intake, those with BPD had a higher weight but lower length-for-age, resulting in higher BMI z score compared to BPD-free infants, thereby suggesting that infants with evolving BPD may require different growth and nutritional targets compared to BPD-free infants.

In infants with evolving lung disease, weight gain may not reliably reflect lean tissue accretion. Pathophysiologically, BPD is characterized by dysregulated lipid metabolism, including a significant deficiency in pulmonary surfactants and the accumulation of pro-apoptotic sphingolipids like ceramide in tracheal aspirates [24,25]. Additionally, a critical association exists between low circulating levels of essential long-chain polyunsaturated fatty acids, such as docosahexaenoic acid (DHA) and arachidonic acid, and an increased risk of developing the disease [26,27]. Under inflammatory or hypoxic stress, growth can shift toward relatively greater fat accretion or reflect fluid changes, while lean mass accretion may be constrained. Systemic CS may further accentuate catabolism, potentially worsening protein breakdown and limiting lean mass gains even when caloric intake appears adequate.

Anthropometric analyses reveal a significant positive association between the pulmonary score (PS) and the weight-for-length (W/L) z-score in preterm infants [28]. Clinical data demonstrate that as the W/L z-score or percentile increases, pulmonary morbidity worsens. Notably, the high W/L ratios observed in this population are primarily driven by higher weight-for-age rather than length restriction. This imbalance is clinically relevant because severely affected lungs may lack the metabolic capacity to support excessive body mass, leading the BPD Collaborative to recommend maintaining the weight-for-length at the 50th percentile to optimize pulmonary outcomes [29]. Linear growth (length velocity) may be impaired through endocrine disruption (ex. IGF-axis effects) and reduced capacity to translate nutrient intake into tissue growth during physiologic stress. As a result, weight, length, and head circumference trajectories may diverge during and after CS exposure and should be interpreted by domain rather than by weight alone.

The administration of postnatal systemic CS, a particularly dexamethasone, significantly accentuates these catabolic disturbances through intense protein wasting [21,30]. Studies have documented a sharp rise in blood urea concentration within 48 h of initiating dexamethasone, rising from a mean of 2–3 mmol/L to 7.1 mmol/L, which represents a nitrogen retention deficit of approximately 158–170 mg/kg/24 h [31]. This catabolic response is characterized by a marked increase in protein breakdown rather than a decrease in synthesis [32]. Furthermore, a substantial increase in the urinary 3-methylhistidine to creatinine ratio, from a mean of 46 to 77, confirms the accelerated loss of skeletal muscle tissue [31].

To mitigate these catabolic effects, nutritional strategies should prioritize maintaining weight-for-length within the infant’s individualized growth trajectory and providing prolonged, tailored caloric fortification through term, which has been shown to support lean mass recovery. This approach can be guided and closely monitored by a nutrition specialist. Clinicians must also specifically address the significant nitrogen retention deficits and intense protein wasting induced by CS to ensure that nutrient intake promotes functional tissue growth rather than excessive fat accretion.

Beyond acute catabolism, systemic CS induce a “shift to the right” in growth velocity curves, indicating a significant delay in reaching maximal growth velocity for weight, height, and head circumference [16]. CS use has resulted in diminished weight gain and impaired head growth [30,31]. Skinner et al. found that weight gain was significantly lower during treatment (13.2 g/day vs. 30.0 g/day), even when caloric intake was controlled [33]. Paradoxically, despite lower overall weight gain, CS may shift accretion toward increased fat production and reduced protein synthesis, resulting in a higher energy content in newly accreted tissue compared to untreated infants [34]. These effects are further compounded by CS-induced lipid intolerance, hypertriglyceridemia, and glucose intolerance with elevated insulin levels, which collectively impair cellular energy utilization [35,36].

Ultimately, while the catabolic consequences of systemic postnatal CS use are well-documented, there remains a critical research gap regarding the specific growth trajectories of extremely preterm infants managed with modern, targeted low-dose dexamethasone regimens initiated beyond the first week of life.

#### 2.2.2. Neurological Development

CS exert their effects on the developing brain by binding to widely distributed CS receptors, which can suppress neurogenesis, inhibit growth factors, and facilitate apoptosis in vulnerable regions such as the hippocampus. These biological disruptions can lead to delayed myelination and reduced brain volumes, though the ultimate clinical impact is heavily influenced by the dosage and timing of exposure.

The 2021 Cochrane systematic review update on systemic CS initiated within the first seven days of life [37] confirms that early dexamethasone administration significantly reduces the incidence of BPD at 36 weeks’ postmenstrual age. However, this update also reaffirmed significant long-term safety concerns, including a nearly twofold increase in the risk of cerebral palsy and major neurosensory disability among survivors. A more recent 2025 meta-regression analysis by Doyle et al. [9]. offers a nuanced perspective by demonstrating that the association between dexamethasone and survival free of cerebral palsy is dependent on the baseline risk of BPD. Their findings indicate that dexamethasone is associated with improved survival without cerebral palsy when the population risk of BPD exceeds 70%, whereas it is associated with clear harm at a baseline BPD risk below 30%. Importantly, this meta-regression found no strong evidence that these treatment effects differed based on whether dexamethasone was initiated in the first postnatal week or later. As such, there may be equipoise for BPD [38]. On the other hand, hydrocortisone use has not been consistently correlated with adverse impacts on the preterm neonatal brain [39].

Multiple mechanisms have been proposed to explain how CS affect brain development in preterm infants. Antenatal CS are administered in pregnancies at risk of preterm delivery to help accelerate lung maturation [40]. However, early animal studies have shown associations between antenatal CS exposure with deleterious and long-lasting effects on brain development, including significant reductions in total brain weight and volumes [41,42,43,44]. Studies in rhesus monkeys and sheep demonstrate that repeated courses can reduce brain weight by as much as 21% and lead to a 30% reduction in hippocampal neurons [42]. These CS are particularly neurotoxic to the hippocampus, causing neuronal degeneration in the CA1-CA3 fields and dentate gyrus through the inhibition of growth factors and the facilitation of apoptosis [45,46]. Additionally, repeated exposure delays myelination in structures like the corpus callosum and reduces the density of myelin basic protein-immunoreactive oligodendrocytes [47]. Both single and repeated courses of antenatal CS have been linked to astrogliosis, an increase in astrocyte density in white matter, which suggests underlying brain injury even in the absence of overt infarction [47].

As for postnatal CS, early dexamethasone use in preterm pup rats has been shown to cause apoptosis of neural progenitor cells in the hippocampus mediated by CS, specifically glucocorticoid (GC) receptors [48]. GCs cross the blood-brain barrier and bind widely distributed GC receptors, which can suppress neurogenesis and synaptic plasticity [49], whereas mineralocorticoid receptors are more concentrated in the hippocampus and actually support neuronal survival [50].

In a prospective cohort study involving 172 preterm neonates with serial MRI examinations, postnatal exposure to hydrocortisone or dexamethasone was significantly associated with an 8% (1.88 cm^3^) and 10% (2.31 cm^3^) reduction in cerebellar volume by term-equivalent age, respectively, while antenatal betamethasone and overall cerebral volume were not adversely affected [51].

Other earlier studies have associated high, early doses of dexamethasone [52] with reduced total brain and cerebellar volumes and later white matter deficits suggesting dose- and timing-dependent risks (*n* = 7, most received CS within the first 14 days of life, median dose of 0.25 mg/kg/day–0.19–0.90 mg range). In contrast, more recent studies [53] using lower or delayed dosing regimens (*n* = 41, initiated at a median of 36 weeks PMA; total 0.89 mg/kg) have not consistently demonstrated these structural differences. Studies suggest that these treatments, when initiated after the first week of life, do not significantly increase the risk of long-term neurodevelopmental impairment (NDI). For example, in a prospective, randomized study of 59 extremely preterm infants (≤27 weeks’ gestation), who remained on ventilatory support at day 10–21 of life, a 42-day dexamethasone tapering course (7.56 mg/kg total dose) resulted in a significantly higher intact survival rate (normal neurologic examination, IQ > 70, and functioning in school without supplemental educational support) at 7 years of age (75% vs. 34%, *p* < 0.005), compared to a 9-day tapering protocol (4.04 mg/kg total dose) [54].

In comparing effects on neurodevelopment between CS, two landmark randomized controlled trials and their follow-up studies have yielded pertinent data; the DART study (evaluating late (>7 days of life) dexamethasone use) [55,56] and the STOP-BPD study [57,58] (evaluating late (>7–14 days of life) hydrocortisone use). The DART follow-up study evaluated the long-term effects of the DART regimen in chronically ventilator-dependent very preterm infants after their first week of life [56]. Despite being terminated early due to recruitment difficulties, the available data indicated that low-dose dexamethasone was not associated with increased long-term harm regarding neurosensory outcomes, growth, or hospital readmissions. The rate of major disability was 41% in the dexamethasone group compared to 31% in the placebo group, and the incidence of cerebral palsy 14% in the dexamethasone group and 22% in the placebo group (22%), though these differences were not statistically significant.

The STOP-BPD (Systemic Hydrocortisone Initiated 7 to 14 Days After Birth) trial [57], was designed to evaluate whether systemic hydrocortisone could improve respiratory outcomes without inducing the neurodevelopmental harm seen with historical dexamethasone regimens. Follow-up on this STOP-BPD study population [57,58] found no significant difference in the composite outcome of death or NDI at 2 years’ corrected age compared to a placebo. Specifically, the rate of NDI was 43.9% in the hydrocortisone group versus 46.5% in the placebo group. Individual domains, including cognitive and motor scores on the Bayley Scales of Infant and Toddler Development, as well as the incidence of cerebral palsy, showed no statistically significant differences between the groups.

Both of these long-term follow-up studies (STOP-BPD and DART) are quite different from historical studies where CS were initiated very early (within the first week of life), which were sometimes linked to higher rates of cerebral palsy [59]. Both the DART and STOP-BPD studies focused on infants at high risk for BPD who received treatment later in their clinical course. The DART study investigators noted that for infants with a very high baseline risk of chronic lung disease, the observed neutral or potentially beneficial effects on the combined outcome of death or cerebral palsy were consistent with meta-regression models [56].

Interestingly, a 2025 prospective cohort study including 392 preterm infants (≤32 weeks GA) used magnetic resonance imaging and demonstrated that low-dose dexamethasone initiated after the first postnatal week for evolving BPD was associated with larger cerebellar and subcortical grey matter volumes at term-equivalent age, as well as improved motor scores at two years, in multivariable analyses. These findings suggest potential neuroprotective benefits rather than harm in the highest risk infants [53]. Additionally, a recent trial randomized preterm newborns who had previously been intubated to either hydrocortisone or placebo at 14–28 days of life [60]. At 2 years corrected age, 84/330 (26%) of hydrocortisone exposed and 71/329 (22%) of placebo exposed children had cerebral palsy, which was not statistically different. In addition, the rate of abnormal Bayley III composite scores were not different between the groups.

This risk-stratified framework aligns with observations that the balance between neurotoxicity and neuroprotection may shift with increasing BPD severity, underscoring the importance of considering underlying disease biology when evaluating long-term neurodevelopmental effects of postnatal CS exposure.

#### 2.2.3. Cardiopulmonary Integrity: Drivers of Metabolic Capacity and Anabolic Potential

Beyond their direct effects on somatic proportions, postnatal CS play a complex role in the maturation and functional integrity of the pulmonary and cardiovascular systems, which serve as the primary physiologic drivers of an infant’s overall metabolic capacity and anabolic potential.

Concerns have been raised regarding CS and impaired pulmonary development [61]. Studies have shown associations between postnatal CS use and transient growth arrest in the lungs, potentially contributing to exacerbated pulmonary hypoplasia [62].

Additionally, both BPD severity and CS use have been associated with adverse impacts on the cardiovascular system and the myocardium itself [63,64,65]. In Parts 1 and 2 of this review series, we describe in more detail the respiratory and cardiovascular impacts of CS on infants with evolving BPD.

Ultimately, all systems (including the pulmonary and cardiovascular) dictate the physiological environment and metabolic capacity for tissue accretion, directly influencing an infant’s overall anabolic potential and the quality of somatic development.

In all, evidence suggests that in infants at higher baseline risk of BPD, systemic CS may confer net protective effects when underlying disease-related mechanisms are considered. Evolving BPD is characterized by persistent pulmonary inflammation, recurrent hypoxic episodes, and sustained respiratory insufficiency, all of which may adversely affect cerebral growth and integrity through inflammatory injury, impaired oxygen delivery, and increased metabolic stress. In this context, prolonged respiratory morbidity may itself represent a significant driver of adverse neurodevelopmental outcomes. By attenuating pulmonary inflammation and improving lung mechanics and oxygenation, dexamethasone may reduce the cumulative burden of hypoxia and systemic catabolism in these high-risk infants, thereby indirectly supporting cerebral development. This risk-stratified framework aligns with observations that the balance between neurotoxicity and neuroprotection may shift with increasing BPD severity, underscoring the importance of considering underlying disease biology when evaluating long-term neurodevelopmental effects of postnatal CS exposure. This finding derives from meta-regression analyses and reflects population-level baseline risk; it should not be interpreted as an individual bedside cutoff. Instead, it supports risk-stratified decision-making when weighing potential benefits and systemic trade-offs.

Going forward, we need to understand how individualized dosing and timing of dexamethasone based on a newborn’s underlying risks for BPD will impact neurodevelopment. The varying impacts of postnatal CS timing and duration on neonatal growth trajectories, comparing historical long-duration protocols with modern shorter courses, are summarized in Table 1.

#### 2.2.4. Skeletal Health

Metabolic bone disease of prematurity (MBDP) is a multifactorial disorder marked by bone demineralization, frequently affecting very low birth weight (<1500 g) infants [68]. Numerous antenatal and postnatal risk factors contribute to its development including postnatal CS exposure [68]. Two retrospective cohort studies including 109 and 856 preterm infants respectively, found that postnatal CS exposure and dosage is associated with MBDP [69,70].

Additionally, a study involving nine premature infants (<32 weeks) treated with tapering doses of dexamethasone (0.5–0.1 mg/kg/day) for chronic lung disease found that during hospitalization, they experienced significantly lower mean rates of bone mineral accretion in the distal radius (measured by single photon absorptiometry) and reduced plasma phosphorus levels [71]. The same study also indicated that dexamethasone therapy compromised growth, noting significantly lower rates of weight, length, and head circumference growth, with catch-up linear growth not evident by 6 months corrected age.

Ultimately, the evidence suggests that postnatal CS exposure, which has been associated with increased MBDP risk, can alter bone mineral accretion and depress overall linear growth in vulnerable premature infants.

#### 2.2.5. Gastrointestinal Integrity

Postnatal CS use has also been linked to a higher risk of gastrointestinal perforation, more specifically hydrocortisone when used concomitantly with non-steroidal anti-inflammatory drug (NSAID) therapy such as indomethacin [72]. In fact, one of the first multicenter randomized trials of prophylactic hydrocortisone was prematurely terminated at 360 neonates due to this concerning finding of increased intestinal perforation in infants treated both with hydrocortisone and indomethacin [73,74]. However, in the absence of early indomethacin, early (<7 days) low-dose hydrocortisone (1 mg/kg/day for 9 days followed by 0.5 mg/kg/day for 3 days) has not been associated with an increased incidence of spontaneous gastrointestinal perforation [73].

Consistent with these findings, the PREMILOC trial [75], which utilized a similar early, low-dose hydrocortisone protocol, found no increased risk of gastrointestinal perforation. Furthermore, real-world evidence from a Swedish cohort [76] confirms this safety profile, reporting no significant differences in the incidence of spontaneous intestinal perforation or necrotizing enterocolitis between infants exposed to the PREMILOC regimen and those who were not.

In addition, spontaneous perforation of the gastrointestinal tract has been associated with concomitant indomethacin and higher-dose dexamethasone therapy given within the first week of life [77,78]. This complication has not been observed with lower-dose dexamethasone (0.15 mg/kg/day for 9 days followed by a tapering dose over 7 days) regimens initiated after the first week of life [78]. In extremely preterm infants, increased risk of spontaneous intestinal perforation appears most consistently linked to specific co-exposures and timing; all potentially contributing to mucosal damage (enzyme activity and bacterial overgrowth) underscoring that risk attribution to CS alone may be misleading without detailing concomitant medications and timing of exposure [79,80,81].

Evidence regarding perforation risk with later (>7 postnatal days) systemic CS exposure is less consistent and should be interpreted cautiously. Where possible, studies should be contextualized by CS type, dosing, timing, and co-medications. While physiologic CS levels have been documented to protect against such injury in adults [82], in extremely preterm infants, CS may instead promote mucosal maturation that predisposes to perforation [83,84], as evidenced by studies linking dexamethasone to altered intestinal growth factor profiles and structural changes, and by observations of higher cortisol levels in infants with spontaneous perforation [74]. Additionally, it is proposed that CS may inhibit neuronal nitric oxide synthase, while indomethacin suppresses endothelial nitric oxide synthase, together potentially impairing intestinal motility [85].

#### 2.2.6. Impacts on the HPA Axis

Cortisol is essential for modulating inflammation, a key driver of BPD pathogenesis [86]. Fetal cortisol production is negligible before 23 weeks’ gestation due to immaturity of the HPA axis and rises substantially only after approximately 30 weeks’ gestation [87]. Consequently, infants born extremely preterm have limited capacity to maintain homeostasis when exposed to stressors such as supplemental oxygen, infection and mechanical ventilation [88]. The resulting insufficient serum cortisol is known to contribute largely to the inflammatory BPD phenotype and some extremely preterm infants fail to mount an adequate cortisol response to stressful events. Cortisol precursors, corticosterone and 17-hydroxy-progesterone, have been shown to be elevated in extremely preterm newborns indicating an immature steroidogenic pathway consistent with this impaired adrenal response [89,90,91,92,93,94,95]. In addition, many preterm infants exhibit relatively high cortisol concentrations yet remain unable to meet increased physiological demand, increasing the risk of adrenal crisis [89].

Additional determinants of circulating cortisol include the physiologic postpartum decline, prior exposure to antenatal CS and exposure to postnatal CS [96]. Additionally, the absence of a diurnal rhythm in neonates limits the reliability of morning cortisol measurements commonly used in older populations. Short courses of dexamethasone or betamethasone (<10 days) either pre- or postnatally carry little risk of neonatal adrenal suppression [97]. However, prolonged postnatal systemic CS therapy (>1–2 weeks) has been shown to suppress the HPA axis and produce iatrogenic adrenal insufficiency that may persist for weeks following discontinuation [98]. The Canadian Pediatric Society notes that while symptomatic adrenal suppression, including adrenal crisis, is rare, it remains a serious risk of systemic and inhaled CS therapy. However, when used per guidelines, inhaled CS and short systemic courses seldom cause clinically significant adrenal suppression. In addition, stress-dosing (with hydrocortisone) is recommended during critical illness or major surgery for children currently on CS, and should be considered for up to a year after discontinuation unless normal HPA axis function is confirmed [99]. Interestingly, prophylactic administration of low-dose hydrocortisone has been shown to protect against functional adrenal insufficiency in cases of extreme prematurity, while also reducing the damaging impact of inflammation on lung development [100]. Although, some data exists regarding the impacts of the use of exogenous CS on adrenal insufficiency in infants and children (due to the wide use of chronic CS for children with asthma) [101], little work has described specifically the short and long-term impacts of systemic/inhaled CS on adrenal function of preterm neonates [98]. As such, improved strategies for systematic evaluation of how contemporary postnatal CS regimens for BPD alter adrenal function are required to optimize and individualize dosing in this vulnerable population. Given variable recovery time after prolonged systemic exposure, discharge planning should include family education regarding stress-dosing during major illness/surgery and consideration of follow-up to confirm HPA-axis recovery when clinically indicated.

## 3. Precision Surveillance: Precision Tools for Monitoring Outcomes

**Figure 4:**
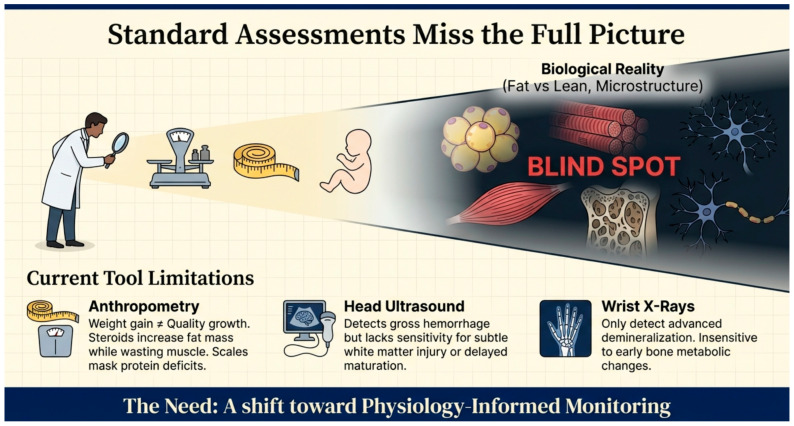
Standard Assessments Miss the Full Picture—Identifying the limitations of current clinical metrics and the need for precision surveillance.

As traditional tools like anthropometry and head ultrasound often miss the “biological reality” of tissue quality and microstructure, Figure 4 illustrates the clinical blind spots that necessitate a shift toward physiology-informed monitoring.

To address the limitations of traditional metrics and provide deeper mechanistic insight into the systemic consequences of corticosteroid exposure, this section introduces a precision surveillance framework of advanced tools, ranging from multi-modal neuroimaging and functional motor assessments to body composition analysis and biochemical markers, designed to monitor an infant’s neurological, somatic, skeletal, and endocrine-metabolic developmental trajectories. For centers lacking advanced resources like MRI or ADP, serial anthropometry interpreted by domain (weight, length, and head circumference) and clinical bedside tools like NIRS provide accessible alternatives for longitudinal tracking.

### 3.1. Monitoring Somatic Growth and Body Composition

Anthropometric measurements, including weight, length, and head circumference, remain the cornerstone of growth monitoring in preterm infants and are routinely collected throughout hospitalization and follow-up. They have been used in countless studies with a wide range of results reported; some that growth following exposure to postnatal CS is stunted and others that it remains unchanged [20,102,103]. Nonetheless, while these measures provide valuable longitudinal data and are easily standardized, anthropometric measures offer only a crude approximation of body composition in CS-treated infants, as they cannot distinguish fat from lean tissue and substantially underestimate fat mass. Although easy to perform, these measurements lack the precision needed for clinically unstable preterm infants and provide limited insight beyond basic growth, falling far short of the detailed structural information required to understand how CS shape early body composition.

One such effort is mid-upper arm circumference (MUAC) measurement, an emerging measurement in assessing nutritional status in this population. MUAC reflects the combined contribution of muscle and fat mass, and in moderately preterm infants, its variation has been shown to be significantly associated with body adiposity [104]. A distinct advantage of MUAC over traditional weight-based metrics is its relative stability during rapid fluid shifts; because the upper arm is less susceptible to changes in fluid status than other body regions, it can provide a more accurate assessment of tissue quality in infants with edema or those receiving CS [104,105]. Furthermore, its simplicity and ease of use make it a practical bedside alternative when accurate length or weight measurements are compromised by medical equipment, clinical instability, or physical contractures [105,106]. While recognized by the Academy of Nutrition and Dietetics and American Society for Parenteral and Enteral Nutrition (ASPEN) as a core indicator for identifying pediatric malnutrition, MUAC remains underutilized in clinical practice, particularly among extremely preterm infants where technical challenges like skin compression during measurement require careful standardization [104].

Assessment of body composition, including the relative distribution of fat and lean mass, provides a more nuanced understanding of growth quality beyond simple weight gain. This distinction is especially relevant following CS exposure, where increases in weight may not correspond to proportional gains in lean tissue or optimal somatic development. Techniques such as Air Displacement Plethysmography (ADP) and bioelectrical impedance analysis (BIA) are leading non-invasive methods for research and growing clinical use. ADP leverages whole-body air pressure displacement to calculate body volume and, from that, fat and fat-free mass [107]. However, its accuracy in infants with lung disease remains uncertain, as the method depends on prediction equations for thoracic gas volume, equations that have not been validated in populations with altered pulmonary physiology [108]. Because lung disease can change the amount of air in the chest, measurements in these infants must be interpreted cautiously, and further research is needed to determine how abnormal lung volumes affect ADP estimates. The devices also cannot be used in clinically unstable infants, including those requiring oxygen therapy, intravenous fluids, or other acute supports, which limits its applicability in many neonates with evolving chronic lung disease who may be receiving systemic CS. In addition, ADP devices are restricted to infants weighing 1–10 kg with a body volume above 1.85 L [109], potentially excluding the smallest and most medically fragile preterm infants, a group that typically includes those receiving dexamethasone.

Further, BIA as a portable, easy-to-use, and practical tool with significant potential, but it ultimately concludes that none of the current BIA equations can be recommended for clinical or research use in infants under 24 months at this time [110]. However, it is limited by its sensitivity to rapid hydration shifts (common in infants with lung disease or receiving dexamethasone), its minimal advantage over simple weight- and length-based estimates in infants under six months, and the high risk of bias and poor methodological quality in studies involving preterm or nutritionally vulnerable populations.

Studies examining postnatal CS use have reported variable effects on growth, with some suggesting transient growth suppression and others indicating later catch-up, underscoring the importance of longitudinal assessment. Interpreting these findings is further complicated by confounding factors including illness severity, nutritional practices, and concurrent therapies.

Despite the centrality of growth outcomes in neonatal research, many studies continue to rely predominantly on weight-based metrics, with limited integration of body composition assessments. Inconsistent timing of measurements and short follow-up periods further restrict the ability to evaluate whether early growth patterns translate into meaningful long-term differences.

A comprehensive evaluation of growth in infants exposed to postnatal CS will require longitudinal monitoring that extends beyond traditional anthropometric measures to include assessments of body composition where feasible. Such approaches are essential to determine whether contemporary CS regimens influence not only the rate of growth, but the quality and sustainability of somatic development in preterm infants. As outlined above, studies examining postnatal CS use have reported variable effects on growth, with some suggesting transient growth suppression and others indicating later catch-up, underscoring the importance of longitudinal assessment.

### 3.2. Monitoring Neurological Maturation

Given the inconsistent evidence regarding neurodevelopmental outcomes and structural brain changes associated with postnatal CS use, it is important to collect robust data on brain development and injury following exposure to lower-dose regimens that are becoming standard practice. The standard modalities for evaluating brain structure and neurodevelopment in preterm infants include head ultrasound, magnetic resonance imaging (MRI) and long-term neurodevelopmental assessments by expert teams using standardized language, cognitive and motor testing [111]. Head ultrasound is widely used at the bedside to detect gross abnormalities such as intraventricular hemorrhage or periventricular leukomalacia, but it lacks sensitivity for more subtle injuries and delayed maturation [112]. In contrast, MRI offers superior resolution and quantitative assessment of brain volumes, cortical folding, and microstructural integrity, making it the gold standard for research and detailed evaluation of preterm brain development. However, although MRI is a powerful modality for assessing structural abnormalities, there is currently insufficient evidence to support its routine use as a clinical screening tool, as findings do not reliably predict long-term outcomes nor necessarily alter management [111,113]. This is consistent with evidence from Hintz et al., which demonstrated that while significant cerebellar lesions on MRI were associated with disability, near-term conventional MRI did not substantively enhance the prediction of severe early school-age outcomes [113]. Consequently, the Canadian Paediatric Society does not recommend routine term-corrected MRI at this time, suggesting it instead be reserved for infants with moderate-to-severe anomalies on head ultrasound, cases where clinical risk for white matter injury is increased, or for parental reassurance when balanced against cost and clinical stability [111]. In this context, MRI may play its most vital role in research by helping to better understand the impact of different treatment strategies and the timing of steroid exposure in relation to other comorbidities

Baseline MRI reference values for regional brain volumes and diffusion metrics have been established in large preterm cohorts, although variation across centers and protocols persists [114,115,116]. Despite MRI’s strengths, most studies examining the effects of postnatal CS have relied on single time-point imaging, often conducted weeks after exposure, leaving uncertainty about how brain structure evolves during treatment itself, and whether these findings truly reflect the diversity of neurodevelopmental outcomes [39,117]. Only a few investigations have paired serial imaging with functional neurodevelopmental assessments [51,118]. Furthermore, much of the earlier work has focused on high-dose regimens given in the first week of life (protocols now largely abandoned) limiting their applicability to modern practice. Whereas, as mentioned above, a recent 2025 study of infants treated with lower-dose dexamethasone initiated after the first postnatal week (more akin to our contemporary clinical standards) reported no adverse macrostructural effects on MRI and even suggested possible neuroprotective benefits for motor development [53].

This highlights how urgently updated longitudinal imaging data are needed to understand whether these newer regimens alter the developmental trajectory in meaningful ways. This knowledge gap will only be addressed through well-designed longitudinal studies that incorporate monitoring strategies such as MRI to comprehensively and longitudinally assess brain development and structure in infants receiving postnatal CS.

While neuroimaging provides critical insight into brain structure, functional neurodevelopmental assessments are essential for capturing the dynamic and often subtle effects of postnatal CS exposure on early brain function and developmental trajectories. These tools allow clinicians and researchers to assess neuromotor organization, cortical activity, autonomic regulation, and behavioral competence during periods when structural abnormalities may be absent or evolving.

Prechtl’s General Movements Assessment is a sensitive early biomarker of neurodevelopmental vulnerability, with evidence suggesting higher rates of abnormal movement patterns and suboptimal motor organization in preterm infants exposed to postnatal CS [119]. In this cohort study including 282 preterm infants who received (*n* = 67; 23.75%) and did not receive postnatal CS, infants underwent the GMA during the fidgety period of infant development (9–20 weeks post-term equivalent age) [119]. Authors highlight the use of the Motor Optimality Score revised (MOS-R) as a detailed scoring system within the GMA as infants receiving postnatal CS were significantly more likely to have a suboptimal score (below 20) than those in the no-CS group (14.8% vs. 2%). Additionally, in 2012 a longitudinal study of 56 infants video recorded GM quality before and after treatment with CS (comparing a hydrocortisone to dexamethasone treated group [120]. Authors note there was no difference in the GM quality between the hydrocortisone and dexamethasone groups until term age; at 3 months hydrocortisone infants had a significantly higher median MOS (25) compared to dexamethasone infants (21). A follow-up study to this one, performed by the same group looked at the effect of low-dose dexamethasone treatment on the quality of GMs, reflecting more contemporary practices [66]. They found that out of 17 infants treated with low-dose dexamethasone neurological functioning improved with the majority having normal neurodevelopment at the age of 12–36 months. Interestingly, when comparing the low-dose dexamethasone infants to those exposed to higher doses, the low-dose infants had higher MOSs at 3 months than high-dose infants even after adjustment for confounders. Additionally, MOSs at 3 months did not differ between low-dose dexamethasone infants and hydrocortisone treated infants. Overall, the authors argue that GMA can reveal neurological dysfunction before structural damage appears on traditional imaging, making it a crucial window into brain health during periods of rapid development when structural changes are ongoing and CS exposure may have effects.

Electroencephalography (EEG), including amplitude-integrated EEG (aEEG), provides real-time assessment of cortical activity and functional brain maturation. These techniques are commonly used in the neonatal intensive care setting to evaluate background patterns, sleep–wake cycling, and dysmaturity, offering a window into neurophysiologic development during and immediately following therapeutic interventions [121,122]. Evidence suggests that combining MRI and aEEG at term-equivalent age can strengthen predictions of later neurodevelopmental outcomes in preterm infants [123,124]. Early aEEG monitoring is also practical in premature infants, and when paired with head ultrasound findings in the first week of life, may improve the ability to identify infants at risk for short-term adverse outcomes [125]. Additionally, infants with BPD show subtle but measurable differences in their aEEG patterns compared with those without BPD at 36 weeks, indicating that aEEG may hold value for understanding how BPD relates to later neurodevelopment [126]. Despite this potential, EEG and aEEG data in infants with evolving BPD who are receiving postnatal CS remain limited; expanding this work could offer important insights into outcome prediction in this vulnerable population.

The Assessment of Preterm Infant Behavior (APIB) focuses on autonomic, motor, state, and attentional regulation, providing a detailed characterization of how preterm infants respond to environmental and physiologic stressors [127]. As postnatal CS may influence neuroendocrine and stress-response systems, behavioral tools such as the APIB offer valuable insight into functional regulation that may not be captured by imaging alone, though this approach has not yet been applied in this specific context.

The Hammersmith Infant Neurological Examination (HINE) is a standardized and widely used neurologic assessment performed in early infancy, offering a structured evaluation of tone, reflexes, posture, and movement patterns [128]. In very low-birth-weight infants, moderate-to-severe BPD (among other clinical factors) has been associated with abnormal HINE scores [129]. The HINE has demonstrated strong predictive value for later neurodevelopmental outcomes and although not yet leveraged in the context of postnatal CS use, can serve as a practical tool for longitudinal follow-up in infants exposed to postnatal CS.

Despite their individual strengths, these neurodevelopmental tools are frequently applied in isolation and at variable time points, limiting their ability to fully characterize developmental trajectories. Few studies focused on the effects of postnatal CS integrate early functional assessments with detailed exposure timing, dose, and concurrent neuroimaging, leaving uncertainty regarding how transient functional changes relate to long-term outcomes.

Addressing these gaps will require well-designed longitudinal studies that combine complementary neurodevelopmental tools, including GMA, EEG/aEEG, behavioral assessments, and standardized neurologic examinations, across critical developmental windows. Integrating these functional measures with neuroimaging and clinical data is essential to determine whether contemporary, lower-dose postnatal CS regimens meaningfully alter neurodevelopmental trajectories in preterm infants.

### 3.3. Assessing Skeletal Integrity

Beyond neurodevelopmental considerations, postnatal CS exposure raises important questions regarding skeletal development and bone health in preterm infants, a population already at heightened risk for impaired bone mineralization. Given the critical period of postnatal bone accretion and the known effects of CS on calcium metabolism and osteogenesis, tools to assess bone density represent an important yet comparatively underexplored outcome domain.

Dual-energy X-ray absorptiometry (DXA) is a useful tool standard for quantifying bone mineral density and content in pediatric and adult populations [130,131]. Although considered a reference standard for assessing bone mineral status, DXA poses several challenges in preterm infants, including the need for transport to the scanner, susceptibility to motion artifacts, and exposure to cumulative radiation [132]. A few studies have used DXA to assess bone density specifically in infants who received dexamethasone [133,134]. One such study followed preterm infants over time and found that those treated with dexamethasone for BPD, when given additional calories until term, showed no differences in bone, fat, or lean mass at term or at three months corrected age compared with preterm infants without BPD [133]. DXA was emphasized as a highly useful method for assessing infant body composition, though the authors noted that it may overestimate fat mass, requires infants to be off respiratory support, and exposes them to low-dose radiation. While anthropometric measures correlated with DXA, they substantially underestimated fat mass, reinforcing DXA’s value as a comparative benchmark rather than an exact measure. However, the trade-off is that there are not always easily accessible reference data for DXA in this age group.

Quantitative ultrasound (QUS), which assesses parameters such as metacarpal speed of sound (mcSOS) and metacarpal bone transmission time (mcBTT), offers a practical alternative given its portability, rapid acquisition time, and lack of radiation exposure [132]. Most existing studies have focused on bone development from admission through approximately 36 weeks’ gestation, with fewer investigations extending measurements over longer follow-up periods. In one cohort of 98 preterm infants, both mcSOS and mcBTT declined during the first six weeks of hospitalization and continued to change during follow-up, with mcSOS decreasing and mcBTT stabilizing by 24 months; growth parameters were significant contributors to these trajectories [132]. However, QUS is not used in clinical practice for many reasons including but not limited to; lack of established, standardized and validated reference data, difficulty in assessing the smallest, sickest infants etc.

Although plain radiography is not suitable for detecting mild MBD [132], targeted imaging such as wrist X-rays, are now being used to identify skeletal abnormalities and signs of rickets (the X-ray diagnosis). Studies have established wrist X-rays as a valuable tool for assessing MBD in very low birth weight infants [135]. While readily available and familiar in clinical practice, radiographs primarily detect relatively advanced changes in bone mineralization and lack sensitivity for early or subtle alterations in bone density.

In the context of postnatal CS therapy, skeletal assessments have most often been secondary or incidental outcomes rather than systematically evaluated endpoints, let alone longitudinally. As a result, there remains limited understanding of whether observed alterations in bone health reflect transient effects of treatment, underlying illness severity, or lasting changes to skeletal development.

Lateral spine X-rays could also serve as a useful tool for evaluating the systemic skeletal impact of treatment. CS are particularly detrimental to trabecular bone, which is initially affected by mechanisms that include increased osteoclast activation and decreased osteoblast function [136]. As the spine is mainly composed of trabecular bone, infants and children receiving these CS face a significantly increased risk of vertebral fractures, which are recognized as a clinical signature of pediatric corticosteroid-induced osteoporosis [137,138]. In the pediatric population, these fractures are frequently asymptomatic and typically occur early in the CS course, often eluding detection in the absence of routine spine imaging [138,139]. This focus on skeletal integrity could provide a more sensitive diagnostic endpoint specifically in high-risk infants.

While DXA provides the most standardized, quantitative data, its practical limitations make it difficult for routine screening of sick, small preterm infants. QUS is increasingly preferred for bedside assessment due to its safety and portability. Wrist X-rays are typically used to diagnose and monitor existing metabolic bone disease, whereas lateral spin X-ray studies are specific for detecting significant demineralization in high-risk infants.

Existing approaches to monitoring bone health in preterm infants are further constrained by heterogeneity in parameters of bone and mineral metabolism, assessment timing, imaging modality, and outcome definitions. Few studies integrate skeletal imaging with detailed CS exposure data, nutritional status (including the use of elemental hypoallergenic formulas), or concurrent therapies known to influence bone metabolism.

Clarifying the skeletal effects of contemporary, lower-dose postnatal CS regimens will require thoughtfully designed longitudinal studies incorporating appropriate tools for bone health assessment. Combining quantitative modalities such as lateral spine X-rays where feasible, with clinically indicated radiographic evaluations may help determine whether postnatal CS meaningfully alter bone mineralization trajectories in preterm infants.

### 3.4. Evaluating Adrenal and Metabolic Function

As outlined above, baseline cortisol levels tend to be lower in preterm infants, but the clinical significance of this finding remains uncertain [140]. Findings from a prespecified secondary analysis of the PREMILOC trial (described above—utilized an early, low-dose hydrocortisone protocol) revealed that baseline serum cortisol concentrations did not predict BPD-free survival in extremely low gestational age neonates treated with hydrocortisone. Notably, cortisol levels exceeding 889 nmol/L (32 µg/dL) were linked to an increased risk of severe intraventricular hemorrhage in treated infants [88]. These results suggest that elevated early cortisol may diminish the benefit-risk balance of prophylactic hydrocortisone in this population [100]. The current gold standard for diagnosing adrenal insufficiency is dynamic testing with cosyntropin, using either low-dose (1 μg IV) or high-dose (125–250 μg IV/IM) protocols, both of which show comparable diagnostic accuracy [141]. Cortisol levels are typically assessed at 0, 30, and 60 min post-stimulation. As cortisol assays advance, traditional thresholds of 18–20 μg/dL (500–550 nmol/L) are being reconsidered, with lower cutoffs (400 nmol/L)now recommended for newer monoclonal antibody-based tests [142,143].

In addition to single cortisol measurements, as mentioned above, monitoring supplementary biomarkers such as 17-hydroxyprogesterone and corticosterone if available could provide valuable insight into adrenal insufficiency and support more informed clinical decision-making [89].

While these diagnostic tools and biomarkers enhance our ability to assess HPA axis function in preterm infants, there remains a critical gap in longitudinal monitoring, before, during, and after CS treatment, which is essential for optimizing safety and personalizing CS therapy.

In addition to the conventional clinical and imaging modalities described above, several emerging biofluid markers have shown promise for monitoring infants’ responses to postnatal CS therapy and guiding individualized treatment decisions. Biofluid markers may complement growth monitoring in CS-treated infants by providing signals of catabolism/anabolism and endocrine effects that can precede visible changes in anthropometry. As discussed above, infants with BPD have been shown to have elevated BNP and NTproBNP levels [63,64,65]. As such, they are being explored as early biomarkers for BPD as groups have shown its levels increase with increased BPD severity. One group has shown that at 14 days of life NTproBNP is higher in those who later develop BPD, interestingly regardless of the presence of a significant PDA [144]. In addition, a study performed in 25 healthy, adult male volunteers observed the effects of a short course of dexamethasone on multiple cardiovascular biomarkers [145]. They found an increase in BNP levels in response to dexamethasone. Even though a variety of studies have attempted to ascertain normal ranges for BNP levels in preterm neonates [146,147,148], they have yet to be described in the preterm population receiving postnatal CS in the context of evolving BPD. Given these observations, BNP and NTproBNP levels could serve as valuable biomarkers to be systematically tracked longitudinally during studies assessing postnatal CS therapy and to elucidate how CS exposure influences cardiovascular stress or to help guide individualized treatment strategies.

Currently, with a variable definition of BPD itself, there is no standard biomarker-based diagnostic strategy for BPD itself. However, there are some research groups making strides aiming to narrow the search to detect BPD earlier. One group has demonstrated the potential for 3 plasma protein markers (sialic acid-binding Ig-like lectin 14 (SIGLEC-14), basal cell adhesion molecule (BCAM), angiopoietin-like 3 protein (ANGPTL-3)) to enable early risk stratification of preterm infants with BPD using proteome screening and enzyme-linked immunosorbent assay (ELISA) in the first week of life [149]. Others have focused efforts in using umbilical cord blood (UCB), urine or tracheal aspirate (TA) samples [150,151]. Additionally explored markers include inflammatory markers, genomic and epigenetic markers, angiogenic growth factors, epithelial factors and more [152]. However, larger, multi-centre clinical trials that incorporate multimodal assessments are needed before these approaches can be applied in clinical practice. Moreover, establishing baseline values for the most promising biomarkers could ultimately help identify meaningful deviations when infants are exposed to postnatal CS; however, this remains an area requiring further research before it can be translated into practice.

### 3.5. Integrative and Emerging Modalities

Another emerging non-invasive monitoring strategy for multi-organ saturation profiles is near-infrared spectroscopy (NIRS) [153]. NIRS may be relevant in CS-exposed infants because tissue oxygenation can reflect physiologic stress and oxygen delivery/utilization, factors that may influence feeding tolerance, anabolic capacity, and growth quality. NIRS are continuous regional monitors providing real-time tissue veinous-weighted oxygen saturations. NIRS is increasingly applied in preterm neonates to monitor regional tissue oxygenation, most extensively in the frontal cerebral cortex, where reference cerebral saturation values are well established and integrated into multiparametric brain monitoring [154,155,156,157,158]. Although NIRS use to assess lung oxygenation is much more recent, preliminary studies demonstrate that pulmonary NIRS reliably reflects changes in lung tissue oxygenation and correlates with arterial blood gases more accurately than pulse oximetry in preterm infants [159,160]. In neonatal respiratory disease, lung regional oxygen saturation measured by NIRS has shown promise for differentiating conditions such as transient tachypnea and pneumonia and correlating with disease severity and respiratory support requirements [161,162]. Importantly, while it is hypothesized that continuous pulmonary NIRS could detect evolving BPD and monitor treatment responses, including to postnatal CS, data specific to this application are lacking. NIRS has the potential to provide a more comprehensive view of oxygen saturation and intermittent hypoxia trends across multiple organs such as the lungs, brain and more, offering a global perspective on the effects of postnatal CS in the neonate. Longitudinal studies and standardized protocols are urgently needed to validate NIRS as a predictive and monitoring tool for BPD and to clarify how evolving lung disease and CS therapy impact pulmonary oxygenation patterns in preterm infants.

Emerging evidence suggests that microRNAs (miRNAs), small non-coding RNAs regulating post-transcriptional gene expression, may play an important role in the pathogenesis and progression of BPD in preterm infants. Prior work has identified differential expression of several miRNAs in tracheal aspirates of preterm neonates with evolving BPD, which has been shown to promote lung injury and fibrosis [163]. In parallel, studies in adults with chronic obstructive pulmonary disease have demonstrated that CS treatment can significantly alter miRNA profiles. Exposure to the CS fluticasone propionate has been linked to modulation of miRNAs involved in inflammatory and remodeling pathways, including miR-320d being identified as a novel mediator of inhaled CS, regulating the pro-inflammatory response of the airway epithelium [164]. However, data has yet to be published surrounding the role of microRNAs in the context of preterm neonates receiving CS postnatally for evolving BPD. As such, it will be important to consider tracking miRNA signatures longitudinally in preterm infants receiving postnatal CS to better characterize their dynamic responses and identify potential biomarkers or mechanistic targets relevant to both efficacy and adverse effects of treatment. A summary of concomitant monitoring tools for postnatal CS effects in preterm infants with evolving BPD is outlined in Table 2.

## 4. A Framework for Physiology-Informed Care

**Figure 5:**
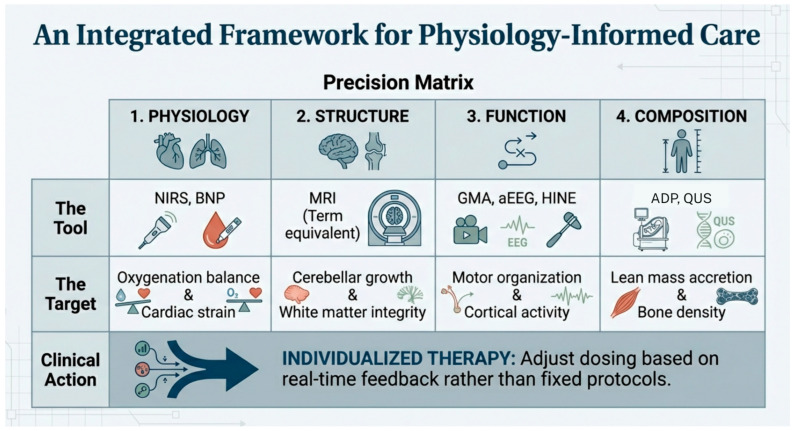
An Integrated Framework for Physiology-Informed Care: A precision matrix to guide individualized therapy through real-time feedback.

An integrated framework for physiology-informed care, categorizing modern monitoring tools into a precision matrix of physiology, structure, function, and composition, is presented in Figure 5. Table 3 provides a summary of CS risks and precision surveillance tools that can be used to monitor each domains state during treatment.

## 5. Conclusions and Directions for Future Research

The evidence surrounding postnatal systemic CS use in extremely preterm infants has evolved from historical high-dose “blast” regimens to contemporary, lower-dose, and later-onset protocols. While uncertainty remains, the current synthesis of evidence provides the following guidance for clinical practice and future investigation:

- Precision Monitoring of Growth: Clinicians should interpret growth by specific domain (weight, length, and head circumference) rather than weight alone, as CS can cause a “shift to the right” in growth trajectories and promote excessive fat accretion over lean mass.
- Implement Domain-Specific Neurological Monitoring:
  ○ Structure: Consider using MRI for quantitative assessment of brain volumes and microstructural integrity.
  ○ Function: Integrate Prechtl’s General Movements Assessment (GMA) with the Motor Optimality Score (MOS-R) to identify neurological dysfunction before structural damage is visible.
  ○ Real-time Activity: Utilize aEEG/EEG to monitor cortical activity and functional brain maturation, especially in infants with BPD.
- Adopt Advanced Somatic and Compositional Tools: To distinguish between lean mass and fat accretion (which CS can shift), consider using Air Displacement Plethysmography (ADP) where feasible, though noting its limitations in unstable infants.
- Prioritize Multi-modal Skeletal Assessment: Since CS compromise bone mineral accretion, clinicians should look beyond plain X-rays to Quantitative Ultrasound (QUS) for bedside safety or Dual-energy X-ray Absorptiometry (DXA) for quantitative bone density. Lateral spine X-rays are specifically recommended to detect asymptomatic vertebral fractures, a “clinical signature” of CS-induced osteoporosis.
- Leverage Physiological and Biofluid Biomarkers:
  ○ Real-time Tissue Oxygenation: Use Near-Infrared Spectroscopy (NIRS) to monitor regional oxygenation in the brain and lungs, which can reflect the infant’s anabolic capacity and response to therapy.
  ○ Cardiac Strain: Track BNP and NTproBNP levels longitudinally to elucidate how CS exposure influences cardiovascular stress.
  ○ Endocrine Stability: Perform Cosyntropin stimulation testing and monitor prohormones (like 17-hydroxyprogesterone) to identify iatrogenic adrenal insufficiency following prolonged CS courses.

The ultimate clinical goal is to integrate these tools into a precision matrix of physiology, structure, function, and composition. This allows for an individualized approach where real-time feedback from these various modalities informs whether to continue, taper, or adjust CS therapy.

The most pressing gap is the validation of these newer tools and the creation of longitudinal datasets that pair these early functional markers with long-term neurodevelopmental outcomes.

## Figures and Tables

**Table 1:** Impacts of postnatal CS timing and duration on neonatal growth trajectories and neurological effects.

Study	CS & Regimen	Age at Initiation & Duration	Impact on Growth	Neurological Effects
Bloomfield et al. (2001) [14]	DEX: 42-day taper vs. 3-day pulse	7 days of life; 42 days vs. 3 days	Mean daily dose significantly influenced daily increase in lower leg length (MDILL; *p* < 0.01). Growth rate drops significantly during treatment.	Higher doses/duration linked to IGF axis suppression (*p* < 0.001). IGF-I and IGFBP-3 levels were independently correlated with leg growth (r^2^ = 0.2, *p* < 0.0001).
DART Trial (Doyle et al., 2006/2007) [55,56]	Low-dose DEX: 0.89 mg/kg total	After 7 days (median 22–24 days); 10 days	No significant difference in weight, height, or head circumference SD scores at 2 years (Weight SD: −0.61 DEX vs. −1.12 Placebo, *p* = 0.30).	No significant increase in harm: Cerebral palsy (CP) rates were 10.3% DEX vs. 11.5% Placebo (*p* = 0.88). “Death or CP” was 22.9% DEX vs. 37.1% Placebo (*p* = 0.19).
Marr et al. (2019) [54]	DEX: 42-day (total ~5 mg/kg) vs. 9-day taper (total ~0.9 mg/kg)	10–21 days of life; 42 days vs. 9 days	Study highlights the shift to shorter regimens to minimize potential harm.	Intact Survival at 7 years: significantly higher in 42-day group (75%) vs. 9-day group (38%) (*p* < 0.01). Survival without neuroimpairment: 93% (42 d) vs. 66% (9 d) (*p* < 0.05).
STOP-BPD (Halbmeijer et al., 2021) [58]	HC	7–14 days of life; 22 days	Survivors at 2 years: Weight < −2 SD was lower in HC group (RR 0.66 [0.51, 0.85], *p* = 0.002).	Death or NDI at 2 years: 56.7% HC vs. 62.7% Placebo (*p* = 0.28). Cognitive scores (mean): 93.4 HC vs. 93.3 Placebo. CP at 2 years: 6.8% HC vs. 6.3% Placebo.
Chandwani et al. (2025) [53]	DEX for evolving BPD (adjusted analysis)	Not specified initiation; TEA MRI	Weight, length, and head circumference were significantly lower in treated infants initially, but adjusted models focused on brain structure.	Adjusted analysis showed DEX correlated with larger cerebellar volume (diff = 0.510, *p* = 0.021) and subcortical grey matter (diff = 0.138, *p* = 0.030) relative to total brain volume.
Tam et al. (2011) [51]	HC or DEX (Observational clinical doses)	During exposure; not specified initiation	General CS exposure significantly associated with decreased growth velocity.	Postnatal HC and DEX linked to 10% reduction in cerebellar volume by TEA. Larger decreases also linked to severe IVH.
Hitzert et al. (2014) [66]	Low-dose DEX: 0.89 mg/kg total dose	After 2nd week of life; 10 days	Shorter mechanical ventilation and later initiation associated with improved movement quality.	Improvement in movements: 69% (9 of 13) of infants with abnormal GMs normalized after treatment (*p* = 0.004). Motor Optimality Scores (MOS) at 3 months were significantly higher than historical high-dose cohorts.
Cochrane Review Meta-Analysis (Doyle et al., 2021) [67]	Systemic DEX or HC; DEX doses typically 0.5–1.0 mg/kg/d initial	Late (≥7 days); duration 3 days to 6 weeks	Meta-analysis of 17 studies showed no substantial differences in childhood growth. Height z-score: MD 0.14 [−0.18, 0.46]. Weight z-score: MD 0.02 [−0.34, 0.38].	Abnormal neurological exams were significantly increased (Typical RR 1.81 [1.05, 3.11]; 4 RCTs). Risk of CP for the DEX subgroup was RR 1.12 [0.79, 1.60] at the latest reported age.
Huysman et al. (2005) [15]	DEX vs. Placebo (DEX + GH study)	Median 2.6–3.1 weeks; 24 days DEX median	Complete growth arrest (including head growth) during the first week of DEX (0.5 to 0.25 mg/kg/d). Weight loss: −3.1 g/kg/day during week 1	Not the primary focus, but noted cystic PVL in 1 placebo infant.
Meta-Regression Analysis (Doyle et al. 2025) [9]	Systemic DEX or HC	Early (1st week) or Late (>7 days)	Not evaluable (Meta-regression focused on the interplay between BPD risk and CP-free survival outcomes).	Association with survival free of CP is BPD-risk dependent: DEX is beneficial when control BPD risk is >70% but associated with harm when risk is <30%. Relationship for DEX: y = −18.6 + 0.374 × BPD risk

Abbreviations: BPD, bronchopulmonary dysplasia; CP, cerebral palsy; DEX, dexamethasone; FMs, fidgety general movements; GH, growth hormone; GMs, general movements; HC, hydrocortisone; IGF, insulin-like growth factor; IGFBP-3, insulin-like growth factor binding protein 3; IVH, intraventricular hemorrhage; MDILL, mean daily increase in lower leg length; MOS, motor optimality score; NDI, neurodevelopmental impairment; PVL, periventricular leukomalacia; TEA, term-equivalent age.

**Table 2:** Summary table of concomitant monitoring tools for postnatal CS effects in preterm infants with evolving BPD.

Monitoring Tool	Clinical Parameters Assessed	Strengths	Limitations
Near- Infrared Spectroscopy (NIRS)	Regional oxygenation of brain, lungs, and other tissues; potential marker of evolving BPD and CS effects	- Continuous, real-time monitoring - Sensitive to early oxygenation changes - Well established in brain monitoring; promising in lung monitoring	- Limited validation for lung application in BPD - Affected by movement and probe placement - No standard thresholds for intervention
Biofluid Markers	Variable depending on marker used; cardiac strain, BPD severity, pulmonary hypertension risk, response to CS.	- Objective biochemical measurement - Early risk stratification potential - Some data linking BNP levels to BPD severity	- Normative ranges poorly defined in preterm infants on CS - Affected by multiple comorbidities (PDA, PH) - Not yet validated for guiding therapy
Micro- RNAs	Gene regulation signatures of inflammation, lung injury, and CS response.	- May reveal mechanistic pathways - Potential biomarkers for treatment efficacy/toxicity - Could help individualize CS dosing	- No neonatal data yet in the context of postnatal CS use - Requires specialized lab capacity - Still investigational and not ready for clinical use
Brain Magnetic Resonance Imaging (MRI)	Brain structure, maturation, microstructural integrity, treatment-related neurotoxicity	- Gold standard for detailed brain assessment - Quantitative and reproducible - Baseline normative datasets exist in preterm infants	- Single time-point imaging limits causal inference - Sedation/logistics in unstable infants - Limited data on lower-dose CS regimens; no consensus on timing/frequency
Computed Tomography (CT) Scan	Acute intracranial pathology (hemorrhage, edema, gross brain injury)	- Rapid detection of acute pathology - Historically used in neonatal neuroimaging	- Inferior sensitivity and specificity compared to MRI - Poor gray/white matter contrast in newborns due to lack of myelination - Exposure to ionizing radiation limits serial use - No identified studies evaluating postnatal CS effects on brain development
Prechtl’s General Movements Assessment (GMA)	Spontaneous movement quality, neuromotor organization, early neurodevelopmental vulnerability	- Highly sensitive early biomarker of neurodevelopmental risk - Detects dysfunction before structural injury appears on imaging - MOS-R allows detailed grading of motor optimality - Responsive to dose-related CS effects	- Requires trained observers and video analysis - Not universally implemented in clinical practice - Functional assessment without direct structural correlation
Electroencephalography (EEG)/Amplitude-Integrated EEG (aEEG)	Cortical activity, functional brain maturation, background patterns, sleep–wake cycling	- Real-time functional assessment - Widely used in NICU settings - Enhances outcome prediction when combined with MRI - Practical for early and repeated monitoring	- Limited data in infants receiving postnatal CS for evolving BPD - Interpretation can be operator-dependent - Functional changes may be transient or nonspecific
Assessment of Preterm Infant Behavior (APIB)	Autonomic, motor, state, attentional, and stress-response regulation	- Captures functional regulation not visible on imaging - Sensitive to neuroendocrine and stress-related effects	- Time-intensive and requires specialized training - Not yet applied specifically to postnatal CS exposure
Hammersmith Infant Neurological Examination (HINE)	Tone, reflexes, posture, movement patterns; early neurologic function	- Standardized and widely used - Strong predictive value for later neurodevelopmental outcomes - Practical for longitudinal follow-up	- Not yet studied specifically in the context of postnatal CS exposure - Less sensitive to subtle early functional changes

**Table 3:** Summary of CS Risks and Precision Surveillance Tools.

Risk Domain	Specific Risks from CS Exposure	Precision Surveillance Tools
Neurological Development	- Hippocampal apoptosis - Reduced regional brain volumes (cerebellum and hippocampus) - Increased risk of CP (associated with early, high-dose protocols)	- Brain MRI - GMA with MOS-R - aEEG/EEG - HINE
Somatic Growth	- Suppression of the IGF-axis leading to “growth arrest” - Reduced weight gain - Impaired linear and head circumference growth	- Detailed growth phenotyping - Serial anthropometric assessments (interpreting trajectories by domain rather than weight alone)
Body Composition	- Induction of a catabolic state with intense protein breakdown (skeletal muscle wasting) - Shift toward excessive fat accretion	- ADP - BIA
Skeletal Health	- Impaired bone mineral accretion - Increased risk of MBDP	- DXA - QUS - Lateral spine X-rays - Wrist X-rays
HPA Axis Function	- Prolonged therapy suppresses the HPA axis, leading to iatrogenic adrenal insufficiency	- Cosyntropin stimulation testing - Supplemental biofluid markers (17-hydroxyprogesterone and corticosterone)
Metabolic & Cardiovascular	- Systemic metabolic shifts including glucose intolerance, hyperinsulinemia, and lipid intolerance - Potential for increased CV strain	- NIRS for real-time tissue oxygenation - Biofluid markers like BNP/NTproBNP
Gastrointestinal Integrity	- Heightened risk of SIP particularly when CS are used concurrently with NSAIDs like indomethacin	- Context-aware Clinical Monitoring (focusing on timing of exposure and pharmacological co-exposures)

Abbreviations: CP (cerebral palsy); MBDP (metabolic bone disease of prematurity); HPA (hypothalamic-pituitary-adrenal); CV (cardiovascular); SIP (spontaneous intestinal perforation); NSAIDs (non-steroidal anti-inflammatory drugs); GMA (General Movements Assessment); MOS-R (Motor Optimality Score revised); CS (postnatal corticosteroids); BPD (bronchopulmonary dysplasia); IGF (insulin-like growth factor); MRI (magnetic resonance imaging); aEEG (amplitude-integrated electroencephalography); EEG (electroencephalography); HINE (Hammersmith Infant Neurological Examination); ADP (air displacement plethysmography); BIA (bioelectrical impedance analysis); DXA (dual-energy X-ray absorptiometry); QUS (quantitative ultrasound); NIRS (near-infrared spectroscopy); BNP (B-type natriuretic peptide); NTproBNP (N-terminal pro BNP).

## Data Availability

No new data were created.

## Abbreviations

The following abbreviations are used in this manuscript:

ADP	Amplitude-integrated electroencephalography
aEEG	Air Displacement Plethysmography
ANGPTL-3	Angiopoietin-like 3 protein
APIB	Assessment of Preterm Infant Behavior
BCAM	Basal cell adhesion molecule
BIA	Bioelectrical impedance analysis
BNP	B-type natriuretic peptide
BPD	Bronchopulmonary dysplasia
CIHR	Canadian Institutes of Health Research
CT	Computed Tomography
DART	Dexamethasone: A Randomized Trial
DXA	Dual-energy X-ray absorptiometry
EEG	Electroencephalography
ELISA	Enzyme-linked immunosorbent assay
FRQS	Fonds de recherche du Québec-Santé
GC	CS
GH	Growth hormone
GMA	Prechtl’s General Movements Assessment
HINE	Hammersmith Infant Neurological Examination
HPA	Hypothalamic-pituitary-adrenal
IGF	Insulin-growth factor
IGF-I	Insulin-like growth factor I
IGFBP-3	Insulin-like growth factor binding protein-3
IV	Intravenous
MBD	Metabolic bone disease
MBDP	Metabolic bone disease of prematurity
MC	Mineralocorticoid
mcBTT	Metacarpal bone transmission time
mcSOS	Metacarpal speed of sound
miRNA	MicroRNA
MOS	Motor Optimality Score
MOS-R	Motor Optimality Score revised
MRI	Magnetic resonance imaging
NDI	Neurodevelopmental impairment
NICU	Neonatal intensive care unit
NIRS	Near-infrared spectroscopy
NTproBNP	N-terminal pro BNP
PDA	Patent ductus arteriosus
PH	Pulmonary hypertension
PMA	Post-menstrual age
PNS	Postnatal CS
PREMILOC	Trial to Prevent Bronchopulmonary Dysplasia in Very Preterm Neonates
QUS	Quantitative ultrasound
SIGLEC-14	Sialic acid-binding Ig-like lectin 14
STOP-BPD	Systemic Hydrocortisone Initiated 7 to 14 Days After Birth trial.
TA	Tracheal aspirate
UCB	Umbilical cord blood
